# Valid ICD-11 PGD Scales and Structured Clinical Interviews Needed

**DOI:** 10.3389/fpsyg.2020.01120

**Published:** 2020-05-28

**Authors:** Maja O'Connor, Lene Larsen, Biretha V. Joensen, Paul A. Boelen, Fiona Maccallum, Katrine Komischke-Konnerup, Richard A. Bryant

**Affiliations:** ^1^Unit for Bereavement Research, Department of Psychology, Aarhus University, Aarhus, Denmark; ^2^The Danish National Center for Grief, Copenhagen, Denmark; ^3^Department of Psychology, Utrecht University, Utrecht, Netherlands; ^4^ARQ National Psychotrauma Centre, Diemen, Netherlands; ^5^School of Psychology, University of Queensland, St Lucia, QLD, Australia; ^6^School of Psychology, University of New South Wales, Sydney, NSW, Australia

**Keywords:** prolonged grief disorder, ICD-11 PGD, psychometrics, PGD scales, valid clinical cut off, structured clinical ICD-11 PGD interview, diagnostic test accuracy

## Introduction

### Official Diagnostic Criteria for Prolonged Grief Disorder Are Coming

In 2022, when the 11th version of the World Health Organization's International Classification of Diseases manual (ICD-11) goes into effect, it will for the first time include diagnostic criteria for Prolonged Grief Disorder (ICD-11 PGD; WHO, 2019). ICD-11 PGD refers to a debilitating grief reaction following bereavement that occurred at least 6 months earlier. It is defined as an intense, persistent, and impairing grief response, characterized by longing for and/or preoccupation with the deceased and intense emotional distress that can include sadness, guilt, anger, denial, blame, difficulty accepting the death, feeling one has lost a part of one's self, an inability to experience positive mood, emotional numbness, or difficulty in engaging with social or other activities. In addition, this response must clearly exceed the grief response expected given the person's cultural and religious context (WHO, 2019). The implementation of ICD-11 PGD will provide health professionals worldwide with a diagnosis to facilitate identification and treatment of patients with PGD. As a condition, ICD-11 PGD will likely receive increased attention in research, clinical, and teaching settings alike. The introduction of the ICD-11 diagnosis for PGD should be considered in light of the conceptualization of disordered grief within DSM 5 (American Psychiatric Association, 2013). The two diagnostic formulations have been found to have significant semantic and empirical overlap when both applied with a set number of symptoms to fulfill (Maciejewski et al., 2016; O'Connor et al., 2019). However, unlike ICD-11, the DSM-5 deemed that there was insufficient data to justify introducing a grief-related diagnosis and instead included Persistent Complex Bereavement Disorder (PCBD; Bryant, 2014) in section Discussion, as a condition for further study; the goal being that additional epidemiological and longitudinal studies may inform a formal diagnosis in future iterations of DSM. Scales have been developed to measure PCBD (Lee, 2015, 2018; Boelen et al., 2018a,b) and an adaption of PCBD (including changing the name to Prolonged Grief Disorder) based on contemporary data has very recently been proposed (see American Psychiatric Association, 2020; Boelen and Lenferink, 2020). However, to date, ICD-11 PGD is still the only official diagnosis for PGD, and it is used in most European Health Services, which is why the ICD-11 PGD will be the focus of the present review.

### The State of Current Instruments Measuring Prolonged Grief Disorder

Complicated, problematic, and persistent grief responses have long been of interest to clinicians and researchers (see Killikelly and Maercker, 2018 for a brief review). Over the past two decades, a number of research groups have worked to describe and identify patients who struggle with prolonged and complicated grief responses (e.g., Prigerson et al., 2009; Shear et al., 2011). The work to develop valid scales and clinical interviews for disordered grief, however, is hampered by a lack of consensus regarding the diagnostic criteria (Lenferink et al., 2019). One of the first instruments to measure prolonged grief was the Inventory of Complicated Grief (ICG) developed in 1995 by Prigerson and colleagues to capture putative markers of what was then called Complicated Grief (Prigerson et al., 1995). The ICG has since been revised several times [Inventory of Traumatic Grief (ITG; Prigerson and Jacobs, 2001); Inventory of Complicated Grief Revised (ICG-R; He et al., 2013)], finally evolving into the PG-13 (Prigerson et al., 2009). The PG-13 is a short, easily administered self-report scale that captures most of the core characteristics, including yearning for the deceased and associated symptoms as defined in ICD-11 PGD (Prigerson et al., 2009). The ICG and its successors, particularly ICG-R and PG-13, are the instruments used in most frequently by studies supporting both ICD-11 PGD and PCBD criteria, and are the scales that most closely resembles ICD-11 PGD. Although studies have found these measures of prolonged grief to have sound psychometric properties, including internal consistency, test-retest reliability, and construct, concurrent, convergent and criterion validity (Prigerson et al., 1995; Lichtenthal et al., 2011; Carmassi et al., 2014; Papa et al., 2014; Boelen and Smid, 2017; Boelen et al., 2018a; Pohlkamp et al., 2018), none correspond directly to the ICD-11 PGD definition.

It is worth noting that a 31 item structured clinical interview for persistent grief reactions has been proposed based on the DSM-5's definition of PCBD (Bui et al., 2015). The goal of this instrument was to promote data collection on grief symptoms to inform future diagnostic criteria. However, it does not offer an assessment corresponding to the current ICD-11 PGD definition. Further, the interview was developed with treatment-seeking patients with persistent grief reactions, and thus at this stage does not provide information that discriminates between patients with and without prolonged grief (Bui et al., 2015).

## Assessment of Diagnostic Test Accuracy is Lacking for Current Scales for Prolonged Grief

While current self-report scales as described above may be reliable and valid in terms of capturing grief symptoms associated with functional impairment (Prigerson et al., 1995), cut off scores to distinguish between people who meet the proposed PGD ICD-11 criteria and people who do not, have apparently not been adequately validated. That is, diagnostic test accuracy (DTA; Campbell et al., 2015) may yet need to be established for these scales. DTA is typically assessed in terms of sensitivity (i.e., the ability of a test to detect true positive cases as defined by a reference standard, here the clinical evaluation), specificity (i.e., the ability of a test to detect those who do not have a specific condition; Trevethan, 2017), and ROC curve analysis (see Leeflang, 2014; Campbell et al., 2015). A PGD scale with established DTA would enable estimation of the prevalence of PGD, tracking of the course of the condition, and identification of risk factors for developing PGD across time, studies, and populations. A questionnaire with sound DTA could also serve as a valid screening tool for PGD. In combination with a structured clinical interview, a self-report assessment tool for ICD-11 PGD with valid DTA would facilitate clinical decision-making, including ruling out the presence of a disorder when relevant, and tracking treatment progress.

We were interested in determining the established DTA of self-report measures of prolonged grief, and therefore we conducted a systematic review of the literature. Our questionnaires of interest were the ICG and its successors, the ITG (Prigerson and Jacobs, 2001), ICG-R (He et al., 2013), and the PG 13 (Prigerson et al., 2009). As described above, these measures represent the most psychometrically sound and commonly used measures of PGD in the literature. The reference standard used in our approach was the presence of a diagnosis of PGD or significant clinical impairment related to bereavement as determined by a clinical diagnostic evaluation performed by clinicians. The review was set up in accordance with the PRISMA-DAT Statement (McInnes et al., 2018). We used the following databases: PubMed, Embase, CINAHL, PsychINFO and Web of science, and went back to January 1995, which is the year of the first publication on the ICG (Prigerson et al., 1995). For a more detailed description of the review, see the protocol submitted to PROSPERO (CRD42019138906).

Only one eligible study comparing a self-report scale and a structured clinical interview for prolonged grief was identified in this systematic review. Boelen et al. (2003) examined the psychometric properties of the Dutch version of the ITG (Prigerson and Jacobs, 2001), with groups of treatment-seeking adults who had experienced the loss of a first-degree-relative. To establish caseness of prolonged grief, participants were interviewed using the Traumatic Grief Evaluation of Response to Loss clinical interview (Prigerson et al., 1998). Unfortunately, however, the symptom-duration of this instrument for establishing caseness is 2 months (in accord with consensus criteria for what was then called “traumatic grief”; Prigerson et al., 1999), which is not compatible with the current duration requirement of 6 months in ICD-11 PGD. This means that the obtained questionnaire cut-off score (DTA) is not applicable today. As a result, we therefore conclude, based on our systematic review, that the field lacks studies that determine the DTA for the self-report scales currently used to measure the presence of disordered grief.

### The Issue of Comorbidity

Disturbed grief is often comorbid with other psychological conditions, and most commonly with depression, anxiety, and PTSD (Jordan and Litz, 2014). Accordingly, the issue of differential diagnosis is crucial to determine if a bereaved person may have ICD-11 PGD alone, or is suffering with other possible conditions such as bereavement-related depression or PTSD. This is particularly important because research indicates that whereas symptoms of prolonged grief respond well to grief-focused psychotherapy (Johannsen et al., 2019), they do not respond optimally to psychological or pharmacological treatments that target depression (Shear et al., 2005, 2016). Some of the symptoms of prolonged grief captured by the ICD-11 PGD overlap with depression, including sadness and reduced interest in activities, and so development of standardized measures of ICD-11 PGD need to be conducted along with differential assessments to ascertain the extent to which the measures can differentiate these potentially overlapping conditions.

### The Need for a Structured Clinical Interview

Although self-report scales are appealing because they can be administered easily and cheaply, self-report scales are not the gold standard for assessing a psychiatric condition. Studies indicate modest concordance between self-report scales and structured clinical interviews (Eaton et al., 2000), with self-report scales typically elevating the incidence of rates of a disorder (e.g., Bui et al., 2015). For this reason, advancing the study of disturbed and prolonged grief requires the development of structured clinical interviews that correspond with the ICD-11 PGD criteria. As noted, a structured clinical interview based on PCBD has been proposed (Bui et al., 2015). However, symptoms do not correspond directly with ICD-11 PGD criteria, and the measure awaits validation with a representative sample of bereaved individuals. A structured clinical interview based on ICD-11 PGD criteria is necessary to standardize the assessment of prevalence rates of PGD with accuracy, and self-report scales alone cannot provide this.

## Discussion

ICD-11 PGD is a complex condition. Until now, there have been numerous definitions of disturbed grief, encompassing ICD-11, DSM-5, and other proposals (Boelen and Lenferink, 2020), which has caused confusion for the field (Lenferink et al., 2019). The introduction of the ICD-11 PGD is welcome because it, for the first time, introduces a formal definition by which disturbed grief can be described and studied. However, the advancement of our knowledge of this disorder requires standardized assessment tools that correspond to these criteria. As mentioned above, existing scales have sound psychometric properties and an ability to capture grief reactions with functional impairment (Prigerson et al., 1995; Lichtenthal et al., 2011; Carmassi et al., 2014; Papa et al., 2014; Boelen and Smid, 2017; Boelen et al., 2018a; Pohlkamp et al., 2018), but these scales do not precisely capture ICD-11 PGD and lack evaluation of diagnostic test accuracy. There is a need for both valid structured clinical interviews for ICD-11 PGD and valid self-report scales for ICD-11 PGD with adequate diagnostic test accuracy and a valid clinical cut off. There is much to learn from earlier work on PGD scales. PGD is a young psychiatric disorder, and further revisions of symptoms and duration criteria may come for both DSM and ICD PGD. Therefore, a new scale should include items corresponding closely to ICD-11 PGD and future developments could usefully have structured scales to also index symptoms specified by DSM to allow careful and standardized comparisons of the two definitions of PGD (cf. Lenferink et al., 2019). Earlier scales (e.g., PG-13) have used multiple response formats including frequency and severity based formats. The development of a new scale would benefit from close collaboration with clinical staff working with PGD, to ensure optimal and clinically meaningful item formulations and response formats. The scale should also be tested and revised accordingly in population-based bereaved samples to ensure its representativeness for general bereavement reactions. Finally, a structured clinical interview for ICD-11 PGD must similarly be developed and validated in clinical and non-clinical samples in close collaboration with clinicians. This interview is necessary to ensure diagnostic test accuracy and to define a valid clinical cut off on the self-report scale for use in research and the clinic. Although ICD-11 will not be formally launched until 2022, this work should be commenced now. Development and use of standardized assessment tools will allow for meaningful comparisons across studies in terms of prevalence, course, and treatment outcomes for individuals with ICD-11 PGD. This will both advance our understanding of how to best conceptualize disturbed grief and foster collaborations in our effort to reduce the significant suffering associated with the condition.

## Author Contributions

The paper was mainly written by MO'C and LL supervised by RB. BJ and LL performed the systematic review supervised by MO'C. PB, KK-K, BJ, and FM read and commented the paper several times in the process. All authors approved the final version of the paper.

## Conflict of Interest

The authors declare that the research was conducted in the absence of any commercial or financial relationships that could be construed as a potential conflict of interest.

## References

[B1] American Psychiatric Association (2013). Diagnostic and Statistical Manual for Mental Disorders, 5th Edn. Washington, DC: American Psychiatric Association 10.1176/appi.books.9780890425596

[B2] American Psychiatric Association (2020). Available online at: https://www.psychiatry.org/psychiatrists/practice/dsm/proposed-changes (accessed May 18, 2020).

[B3] Boelen P. A. Djelantik A. A. A. M. J. de Keijser J. Lenferink L. I. M. Smid G. E. (2018a). Further validation of the traumatic grief inventory-self report (tgi-sr): a measure of persistent complex bereavement disorder and prolonged grief disorder. Death Stud. 43, 351–364. 10.1080/07481187.2018.1480546 30015568

[B4] Boelen P. A. Lenferink L. I. M. (2020). Comparison of six proposed diagnostic criteria sets for disturbed grief. Psychiat.r Res. 285:112786. 10.1016/j.psychres.2020.112786 32000105

[B5] Boelen P. A. Lenferink L. I. M. Nickerson A. Smid G. E. (2018b). Evaluation of the factor structure, prevalence, and validity of disturbed grief in DSM-5 and ICD-11. J. Affect. Disord. 240, 79–87. 10.1016/j.jad.2018.07.041 30059938

[B6] Boelen P. A. Smid G. E. (2017). The traumatic grief inventory self-report version (TGI-SR): introduction and preliminary psychometric evaluation. J. Loss Trauma 22, 196–212. 10.1080/15325024.2017.128448830015568

[B7] Boelen P. A. Van Den Bout J. De Keijser J. Hoijtink H. (2003). Reliability and validity of the Dutch version of the inventory of traumatic grief (ITG). Death Stud. 27, 227–247. 10.1080/07481180302889 12703504

[B8] Bryant R. A. (2014). Prolonged grief: where to after diagnostic and statistical manual of mental disorders, 5th edition? Curr. Opin. Psychiatr. 27, 21–26. 10.1097/YCO.0000000000000031 24270486

[B9] Bui E. Mauro C. Robinaugh D. J. Skritskaya N. A. Wang Y. Gribbin C.. (2015). The structured clinical interview for complicated grief: reliability, validity, and exploratory factor analysis. Depress. Anxiety 32, 485–492. 10.1002/da.22385 26061724 PMC4565180

[B10] Campbell J. M. Klugar M. Ding S. Carmody D. P. Hakonsen S. J. Jadotte Y. T.. (2015). Diagnostic test accuracy: methods for systematic review and meta-analysis. Int. J. Evid. Based Healthc. 13, 154–162. 10.1097/XEB.0000000000000061 26355602

[B11] Carmassi C. Shear M. K. Massimetti G. Wall M. Mauro C. Gemignani S.. (2014). Validation of the Italian version inventory of complicated grief (ICG): a study comparing CG patients versus bipolar disorder, PTSD and healthy controls. Compr. Psychiatry 55, 1322–1329. 10.1016/j.comppsych.2014.03.001 24721191

[B12] Eaton W. W. Neufeld K. Chen L. S. Cai G. (2000). A comparison of self-report and clinical diagnostic interviews for depression: diagnostic interview schedule and schedules for clinical assessment in neuropsychiatry in the Baltimore epidemiologic catchment area follow-up. Arch. Gen. Psychiatry, 57, 217–222. 10.1001/archpsyc.57.3.217 10711906

[B13] He L. Wang J. Tang S. Yu W. Qiuyuan X. (2013). Reliability and validity of the inventory of complicated grief-revised. Chin. Ment. Health J. 27, 937–943. 10.3969/j.issn.1000-6729.2013.12.010

[B14] Johannsen M. Damholdt M. F. Zachariae R. Lundorff M. Farver-Vestergaard I. O'Connor M. (2019). Psychological interventions for grief in adults: a systematic review and meta-analysis of randomized controlled trials. J. Affect. Disord. 253, 69–89. 10.1016/j.jad.2019.04.065 31029856

[B15] Jordan A. H. Litz B. T. (2014). Prolonged grief disorder: diagnostic, assessment, and treatment considerations. Prof. Psychol. Res. 45, 180–187. 10.1037/a0036836

[B16] Killikelly C. Maercker A. (2018). Prolonged grief disorder for ICD-11: the primacy of clinical utility and international applicability. Eur. J. Psychotraumatol. 8:1476441. 10.1080/20008198.2018.1476441 29887976 PMC5990943

[B17] Lee S. A. (2015). The persistent complex bereavement inventory: a measure based on the DSM-5. Death Stud. 39, 399–410. 10.1080/07481187.2015.1029144 26057231

[B18] Lee S. A. (2018). Factorial structure of the persistent complex bereavement inventory: testing a hierarchical factor model. Death Stud. 42, 356–361. 10.1080/07481187.2017.1348402 28678673

[B19] Leeflang M. M. G. (2014). Systematic reviews and meta-analyses of diagnostic test accuracy. Clin. Microbiol. Infect. 20, 105–113. 10.1111/1469-0691.12474 24274632

[B20] Lenferink L. I. M. Boelen P. A. Smid G. E. Paap M. C. S. (2019). The importance of harmonising diagnostic criteria sets for pathological grief. Br. J. Psychiatr. 13, 1–4. 10.1192/bjp.2019.240 31718725 PMC8387857

[B21] Lichtenthal W. G. Nilsson M. Kissane D. W. Breitbart W. Kacel E. Jones E. C.. (2011). Underutilization of mental health services among bereaved caregivers with prolonged grief disorder. Psychiatr. Serv. 62, 1225–1229. 10.1176/ps.62.10.pss6210_1225 21969652 PMC3694810

[B22] Maciejewski P. K. Maercker A. Boelen P. A. Prigerson H. G. (2016). “Prolonged grief disorder” and “persistent complex bereavement disorder,” but not “complicated grief,” are one and the same diagnostic entity: an analysis of data from the yale bereavement study. World Psychiatr. 15, 266–275. 10.1002/wps.20348 PMC5032512 27717273

[B23] McInnes M. D. F. Moher D. Thombs B. D. McGrath T. A. Bossuyt P. M. The PRISMA-DTA Group (2018). Preferred reporting items for a systematic review and meta-analysis of diagnostic test accuracy studies: the PRISMA-DTA statement. JAMA 319, 388–396. 10.1001/jama.2017.19163 29362800

[B24] O'Connor M. Lasgaard M. Larsen L. Johannsen M. Lundorff M. Farver-Vestergaard I.. (2019). Comparison of proposed diagnostic criteria for pathological grief using a sample of elderly bereaved spouses in Denmark: perspectives on future bereavement research. J. Affect. Disord. 251, 52–59. 10.1016/j.jad.2019.01.056 30903989

[B25] Papa A. Lancaster N. Kahler J. (2014). Commonalities in grief responding across bereavement and non-bereavement losses. J. Affect. Disord. 161, 136–143. 10.1016/j.jad.2014.03.018 24751321

[B26] Pohlkamp L. Kreicbergs U. Prigerson H. G. Sveen J. (2018). Psychometric properties of the prolonged grief disorder-13 (PG-13) in bereaved Swedish parents. Psychiatr. Res. 267, 560–565. 10.1016/j.psychres.2018.06.004 29982112

[B27] Prigerson H. G. Horowitz M. J. Jacobs S. C. Parkes C. M. Aslen M. Goodkin K.. (2009). Prolonged grief disorder: psychometric validation of criteria proposed for DSM-V and ICD-11. PLoS Med. 6:e1000121. 10.1371/journal.pmed.1000121 19652695 PMC2711304

[B28] Prigerson H. G. Jacobs S. C. (2001). Traumatic grief as a distinct disorder: a rationale, consensus criteria, and a preliminary empirical test, in Handbook of Bereavement Research. Consequences, Coping and Care, eds. Stroeve M. S. Hansson R. O. Stroebe W. Schut H. A. W. (Washington, DC: American Psychological Association Press), 613–647. 10.1037/10436-026

[B29] Prigerson H. G. Kasl S. V. Jacobs S. C. (1998). Traumatic Grief Evaluation of Response to Loss (TRGR2L). New York, NY: Unpublished manuscript.

[B30] Prigerson H. G. Maciejewski P. K. Reynolds C. F. III. Bierhals A. J. Newsom J. T. Fasiczka A.. (1995). Inventory of complicated grief: a scale to measure maladaptive symptoms of loss. Psychiatr. Res. 59, 65–79. 10.1016/0165-1781(95)02757-2 8771222

[B31] Prigerson H. G. Shear M. K. Jacobs S. C. Reynolds C. F. 3rd Maciejewski P. K. Davidson J. R.. (1999). Consensus criteria for traumatic grief. A preliminary empirical test. Br. J. Psychiatry 174, 67–73. 10.1192/bjp.174.1.67 10211154

[B32] Shear K. Frank E. Houck P. R. Reynolds C. F. III. (2005). Treatment of complicated grief: a randomized controlled trial. JAMA 293, 2601–2608. 10.1001/jama.293.21.2601 15928281 PMC5953417

[B33] Shear M. K. Reynolds C. F. Simon N. M. Zisook S. Wang Y. Mauro C.. (2016). Optimizing treatment of complicated grief: a randomized clinical trial. JAMA Psychiatr. 73, 685–694. 10.1001/jamapsychiatry.2016.0892 27276373 PMC5735848

[B34] Shear M. K. Simon N. Wall M. Zisook S. Neimeyer R. Duan N.. (2011). Complicated grief and related bereavement issues for DSM-5. Depress. Anxiety 28, 103–117. 10.1002/da.20780 21284063 PMC3075805

[B35] Trevethan R. (2017). Sensitivity, specificity, and predictive values: foundations, pliabilities, and pitfalls in research and practice. Front. Public Health 5:307. 10.3389/fpubh.2017.00307 29209603 PMC5701930

[B36] WHO (2019). Available online at: https://icd.who.int/browse11/l-m/en#/http://id.who.int/icd/entity/1183832314 (accessed May 18, 2020).

